# Effect of temperature and air pressure on the incidence of Bell's palsy in Hangzhou: a distributed lag non-linear analysis

**DOI:** 10.1038/s41598-023-47570-2

**Published:** 2023-11-22

**Authors:** Junkang Chen, Zhexuan Yu, Wenhui Zhou, Huafeng Cai, Fanyuan Jin, Jinhua Hu, Erhui Yu, Lihua Xuan

**Affiliations:** 1 The First School of Clinical Medicine of Zhejiang, Chinese Medical University, Hangzhou, 310053 Zhejiang China; 2 Department of Acupuncture and Moxibustion, The First Affiliated Hospital of Zhejiang Chinese Medical University (Zhejiang Provincial Hospital of Traditional Chinese Medicine), Hangzhou, 310006 Zhejiang China

**Keywords:** Neurological disorders, Risk factors

## Abstract

The etiology of Bell’s palsy (BP) is currently unknown, and the findings from previous studies examining the association between seasonal or meteorological factors and BP have been inconsistent. This research aims to clarify this relationship by analyzing a larger dataset and employing appropriate statistical methods. Data from 5387 patients with BP treated at Zhejiang Provincial Hospital of Traditional Chinese Medicine in Hangzhou, Zhejiang Province, from May 1, 2018, to June 30, 2023, was gathered. We assessed the temporal distribution of meteorological factors and the incidence of BP across seasons and months. A distributed lag non-linear model was used to further investigate the lagged and overall effects of temperature and air pressure on the onset of BP. The temporal distribution of BP incidence revealed the highest average number of cases occurring in December and the lowest in June. A correlation existed between BP episodes and temperature or air pressure. The model revealed a higher relative risk during periods of low temperature and high air pressure, characterized by a time lag effect. This correlation was notably more pronounced in female patients and individuals in the young and middle-aged groups. Our findings suggest that exposure to low temperatures and high air pressure constitute risk factors for BP development.

## Introduction

Bell's palsy (BP), recognized as idiopathic peripheral facial paralysis, represents the most prevalent form of facial nerve paralysis. Despite extensive research, the complete etiology and pathogenesis of BP remain elusive. Potential pathogenesis includes factors related to the anatomy of the facial nerve, viral infections, local ischemia and inflammation, exposure to cold, and autoimmune responses^1,2^. Viral infections, in particular, have garnered substantial attention as a significant causative factor, supported by pathological evidence^3^. Meteorological factors such as temperature have been found to influence the processes of multiple viral invasions and the body's autoimmune responses^4,5^, implying that meteorological conditions may indirectly impact the development of BP. In our retrospective study of hospitalized patients, over 33.9% exhibited clear signs of wind or cold exposure to the face preceding the onset of the disease. Collectively, these findings lend support to the potential correlation between BP and meteorological factors.

Numerous epidemiological studies have investigated the association between the onset of BP and seasonal or meteorological factors in recent years, but the findings have exhibited inconsistencies. The majority of studies indicate a higher incidence of BP in colder environments or during winter^6–9^, which may be more pronounced in men^10^. However, some studies have reported that the peak incidence does not necessarily coincide with the coldest months of the year^11–13^ or that BP does not exhibit a clear correlation with seasonal variations or specific months^14,15^. These discrepancies may be attributed to regional variations in meteorological factors across different seasons. Therefore, it is imperative to delve further into the relationship between temperature, atmospheric pressure, atmospheric pollutants and other meteorological factors with BP. Some studies found associations between BP incidence and low temperatures coupled with low relative humidity^11^, high wind speeds^14,16^, sustained high atmospheric pressure or rapid temperature fluctuations^17^, wind chill factor^8^, or elevated concentrations of nitrogen dioxide in atmospheric pollutants^18^. Nevertheless, the inclusion of other common meteorological factors in these studies did not yield evidence of the relevance, resulting in divergent conclusions. This discrepancy may be attributed to limitations in previous research, including a limited number of cases, the absence of more precise subgroup analyses, and statistical methods that do not account for lagged effects of meteorological factors. These emphasize the necessity of further research in this field.

To address these uncertainties and provide more conclusive insights, we conducted a retrospective study that rigorously examined temporal and geographical parameters associated with each included BP case. Cases with unclear onset times, uncertain geographic locations, or incomplete data were excluded from our analysis. In addition, we employed a distributed lag non-linear model (DLNM) with cross-basis computations to thoroughly investigate the relationship between essential meteorological factors and the onset of BP.

## Materials and methods

### Study population

The data for the BP patient study were retrieved from the electronic medical records of Zhejiang Provincial Hospital of Traditional Chinese Medicine, strategically situated in Hangzhou City (coordinates: 120.2°E, 30.3°N), the capital city of Zhejiang Province, renowned for its four distinctive seasons. This dataset encompassed individuals diagnosed with BP who sought medical evaluation across various clinical settings, including the emergency room, outpatient clinics and inpatient wards. Data collection spanned from May 1, 2018, to June 30, 2023, exclusively including cases in which BP diagnoses had been conclusively established by clinicians.

To ensure data precision, a stringent data curation process was implemented. Firstly, we excluded cases involving individuals with the same illness but follow-up visits. In instances where patients transitioned from outpatient to inpatient care, preference was granted to inpatient data due to its comprehensive nature, with the concurrent exclusion of redundant outpatient records. Moreover, recognizing that some patients did not promptly seek medical attention or referral to our hospital following the onset of illness, resulting in discordance between onset time and visit time, a meticulous assessment of BP onset time was carried out on a case-by-case basis. Cases marked by uncertain or unknown onset times were judiciously omitted from the dataset. Additionally, we verified the location of outpatients' health insurance and the contact addresses of inpatients, subsequently excluding cases with onset outside the Hangzhou area or marked by incomplete information. In total, our rigorous data curation process yielded a dataset comprising 5387 BP cases, further categorized into 1763 inpatients and 3624 outpatients.

Our study received the ethical nod from the Ethics Committee of Zhejiang Provincial Hospital of Traditional Chinese Medicine (Approval Number: 2023-KLS-193-01). The study was conducted in accordance with the Declaration of Helsinki and followed relevant guidelines and regulations. Due to the retrospective nature of the study and the de-identification and anonymization of patient data within the cohort, informed consent was waived by the Ethics Committee of Zhejiang Provincial Hospital of Traditional Chinese Medicine.

### Exposure assessment

In our analysis of specific meteorological variables, we meticulously selected factors with a high likelihood of demonstrating a robust correlation with the onset of BP. This selection was guided by clinical expertise and previous research findings. Simultaneously, we considered the accessibility of meteorological data to the general populace in their daily lives, emphasizing its practical significance in guiding strategies for disease prevention and post-incidence protection of the facial nerve. Based on these considerations, we ultimately chose to investigate the correlation between the four common meteorological factors: temperature (°C), air pressure (hPa), wind speed (m/s), and humidity (%), and the incidence of BP. The meteorological parameters investigated in this study were obtained from publicly accessible data provided by the China Meteorological Administration for the Hangzhou region. The aggregated data represent the mean derived from eight daily measurements taken at 2:00 AM, 5:00 AM, 8:00 AM, 11:00 AM, 2:00 PM, 5:00 PM, 8:00 PM, and 11:00 PM.

Seasons were categorized based on traditional Chinese festivals. In accordance with the traditional Chinese 24 solar terms, spring initiates with the 'Beginning of Spring' typically between February 3rd and 5th. Summer commences with the ‘Beginning of Summer,’ which generally falls between May 5th and 7th. Autumn initiates with the 'Beginning of Autumn,' commonly around August 7th to 9th, and winter starts with the 'Beginning of Winter,' typically between November 7th and 8th.

### Statistical analysis

We employed the Spearman correlation to examine the relationship between the incidence of BP and meteorological factors. As meteorological effects on health outcomes are frequently reported to be non-linear and delayed, we modelled the meteorological-BP association using DLNM, which is often applied in studies where disease is associated with meteorological factors^19,20^. We incorporated significant variables into this model by analyzing the time-series distribution of meteorological parameters, the number of BP cases, and the results of the Spearman correlation. The final model was constructed as follows:

$$ Y_{t} \sim Quasi - Poisson\left( {\mu_{t} } \right) $$

$$ {\text{log}}\left( {\mu_{t} } \right) = cb\left( {Weather,\;lag} \right) + ns\left( {Day,\;df} \right) + Week + Season $$

where *Y*_*t*_ represents the number of BP cases with daily onset in the city at time t, and *cb*(*Weather, lag*) denotes the cross-basis function of each meteorological factor describing the exposure-lag-response relationship. We defined 4 degrees of freedom (dfs) for the natural cubic spline curves of temperature and air pressure in the exposure dimension and 6 degrees of freedom (dfs) for the lag effect. Referring to the results of similar studies, we established the lag range to 0–21 days to encompass potential delayed effects adequately. To remove the long-term trend associated with BP, we employed a natural cubic spline function with 7 dfs/year. The day of the week is represented by *Week*, while the season of the year was represented by *Season*, thereby controlling for the effects of day of the week and season. The median value of each exposure variable served as the reference for calculating relative risk.

In addition, we performed subgroup analyses to identify potentially susceptible subgroups and to evaluate whether the association between environment and BP varied by age and sex. We conducted sensitivity analyses to assess the robustness of our findings. We adjusted the delay period from 21 to 14 or 28 days, and the results remained consistent (Fig. S1). Data were analysed using R software (version 4.1.0), and *p-*values were two-sided, with *p* < 0.05 considered statistically significant.

## Results

After meticulous verification and exclusion, our study included a total of 5387 cases of BP in the Hangzhou region. Table 1 presents comprehensive clinical characteristics of patients with BP alongside meteorological parameters on the day of onset. Notably, a higher incidence of the disease was observed among male patients, and BP affected individuals across all age groups, with a notable prevalence among young individuals (53.09%). Onset timing did not display significant clustering throughout the week. The daily mean values of temperature, air pressure, humidity, and wind speed in Hangzhou were recorded as 18.64 °C, 1010.99 hPa, 72.63%, and 2.15 m/s, respectively.

**Table 1:** Clinical characteristics of cases with Bell's palsy and descriptive statistics for daily meteorological parameters on the day of the onset (May 1, 2018–June 30, 2023).

Variable	Number (%)
Number	5387
Hospitalisation	1763
Emergency or outpatient	3624
Gender	
Male	2968 (55.10)
Female	2419 (44.90)
Age	
1–17	193 (3.58)
18–39	2860 (53.09)
40–59	1575 (29.24)
60-above	759 (14.13)
Day of week	
Monday	800 (14.85)
Tuesday	736 (13.66)
Wednesday	738 (13.70)
Thursday	769 (14.28)
Friday	826 (15.33)
Saturday	755 (14.02)
Sunday	763 (14.16)
Daily meteorological factors (Mean ± SD)	
The average temperature (℃)	18.64 ± 8.66
The average air pressure (hPa)	1010.99 ± 9.20
The average wind speed (m/s)	72.63 ± 14.10
The average relative humidity (%)	2.15 ± 0.73

**Figure 1:**
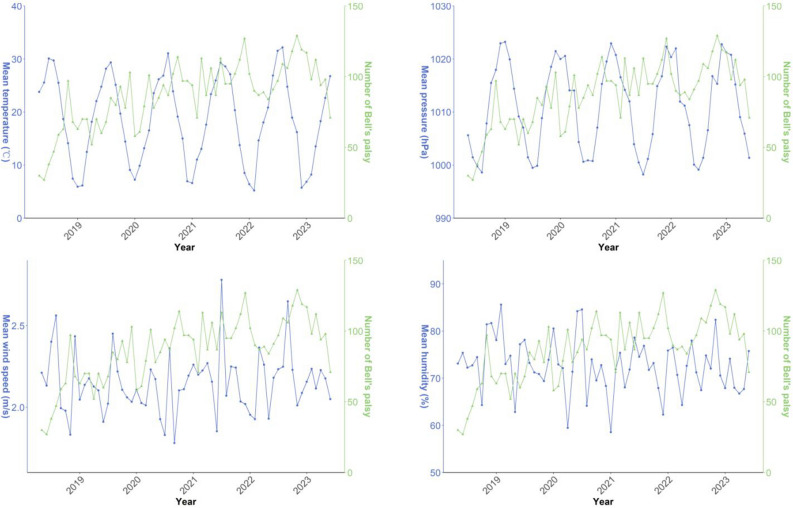
Time series distribution of monthly average meteorological factors and monthly mean number of Bell's palsy cases.

To comprehend the dynamics of meteorological factors and the incidence of BP, we illustrated the time-series distribution of monthly mean meteorological parameters alongside monthly mean number of BP cases throughout the study duration (Fig. 1). The number of patients suffering from BP in our hospital exhibited an upward trend. Temperature and air pressure displayed consistent seasonal oscillations. Additionally, monthly number of BP showed an inverse correlation with average monthly temperature and a positive correlation with average monthly air pressure, while humidity and wind speed exhibited no discernible regular patterns.

**Figure 2:**
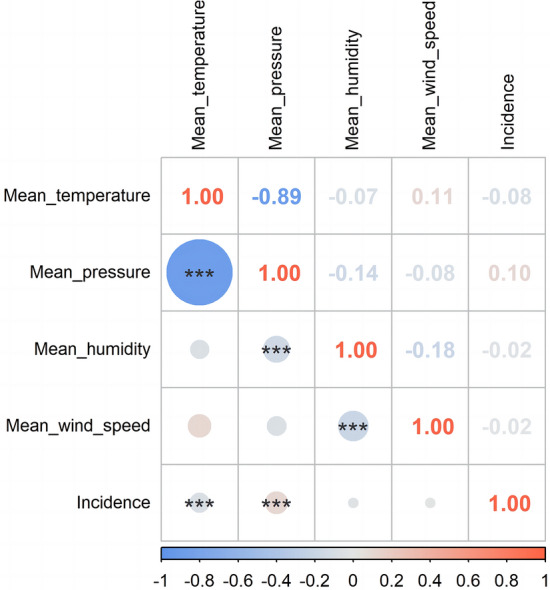
Spearman’s correlation coefficients between the incidence of Bell’s palsy and meteorological factors. (^***^p ≤ 0.001).

Figure 2 displays the Spearman’s correlation coefficients between the incidence of BP and meteorological factors. Temperature is negatively correlated with the incidence of BP, while air pressure is positively correlated with it. Humidity and wind speed exhibit no significant correlation with BP incidence. Additionally, a strong correlation exists between temperature and air pressure (r = − 0.89), while weaker negative correlations are observed between humidity and both air pressure and wind speed.

**Figure 3:**
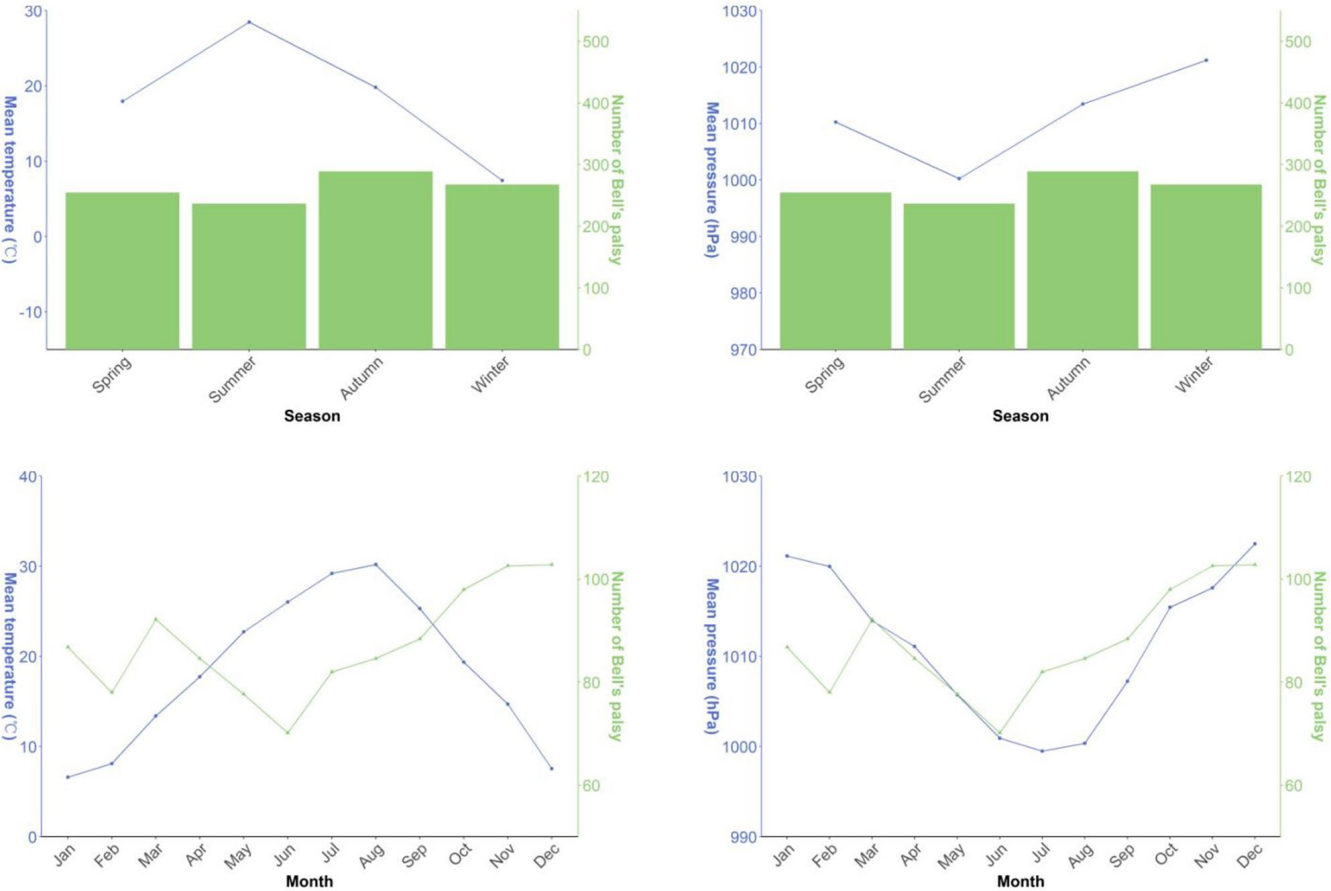
Seasonal and monthly distribution of average temperature, air pressure, and mean number of Bell's palsy cases over a 5-year study period.

Given the influence of various factors on seasonal and monthly variations, Fig. 3 shows seasonal and monthly distribution of average temperature, air pressure, and mean number of BP cases over a 5-year study period. The mean number of BP cases reached its peak during autumn and December, while reaching its nadir in summer and June. Moreover, it exhibited a monthly increase during autumn and winter. Air pressure reached its zenith in winter and December, while attaining its lowest values in summer and July. Conversely, mean air temperature exhibited the opposite trend, with its highest values in summer and June, and its lowest values in winter and January.

**Figure 4:**
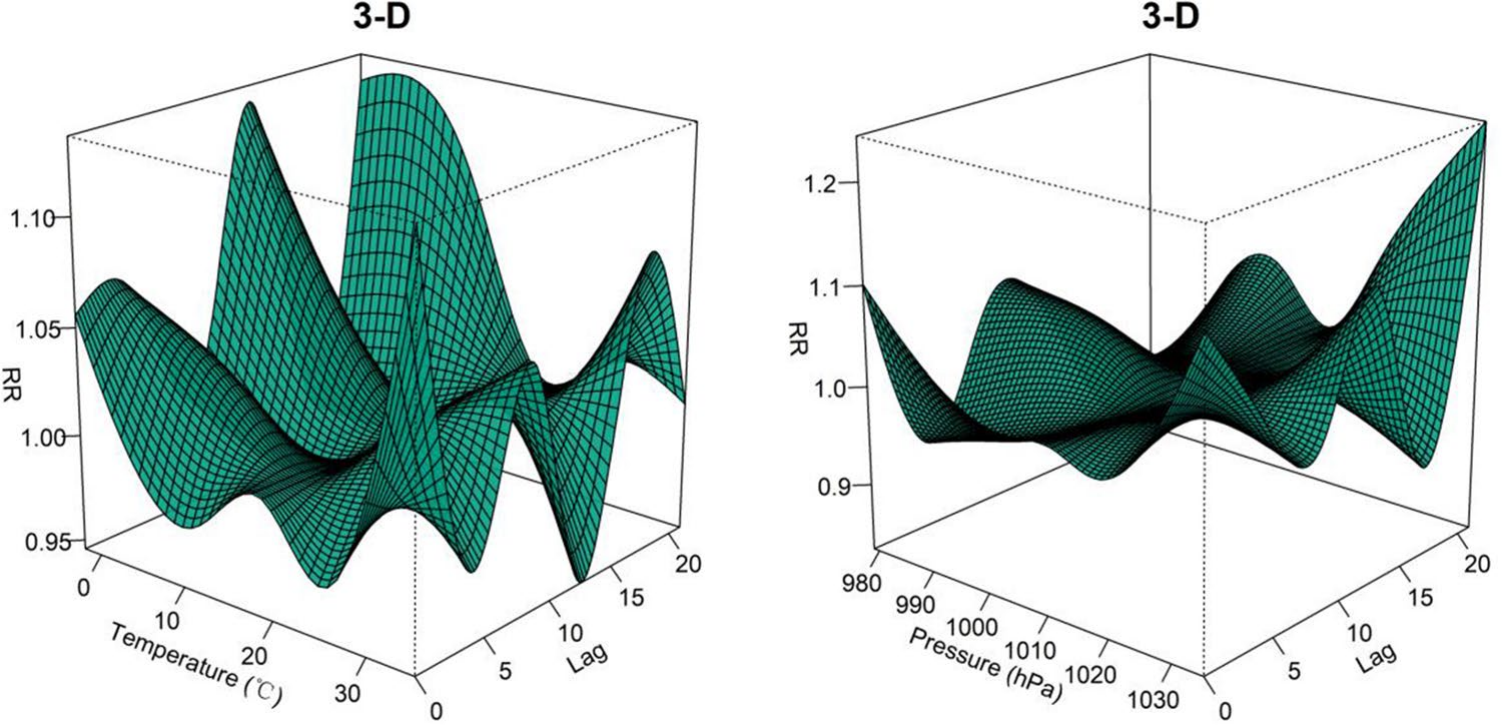
The 3-D plots of the exposure-lag-response association between temperature and air pressure and Bell’s palsy.

**Figure 5:**
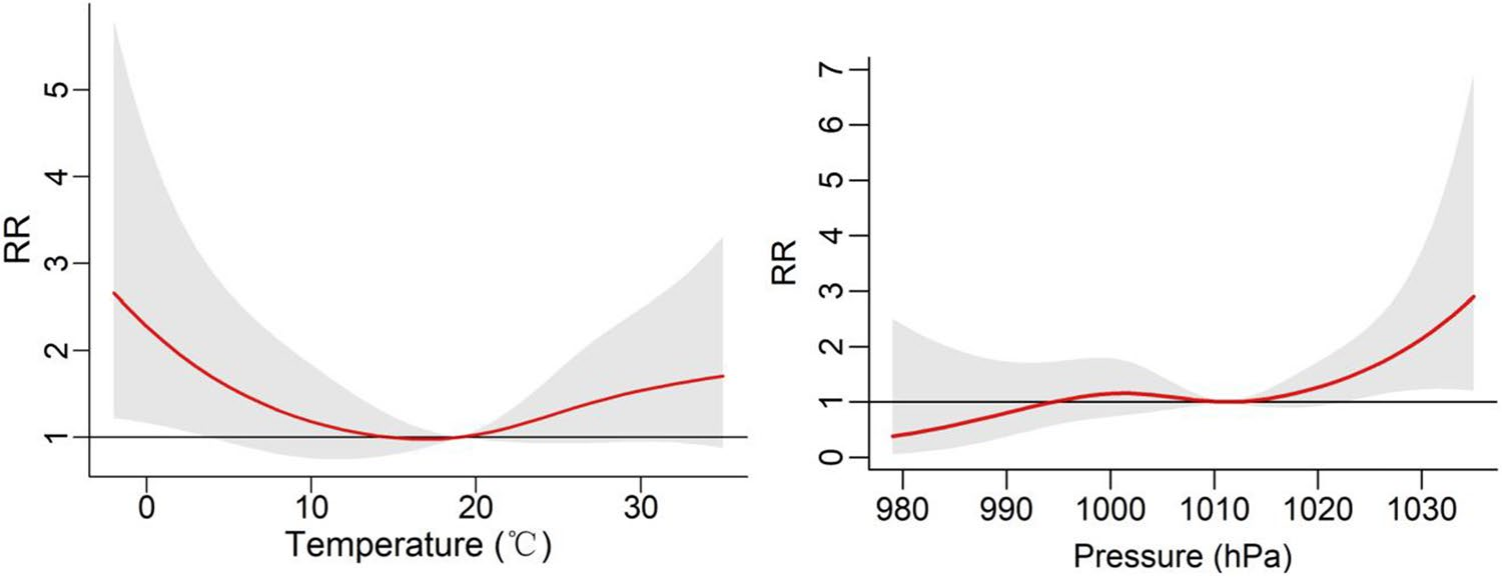
The overall effect of temperature and air pressure on Bell's palsy at lag 0–21.

**Figure 6:**
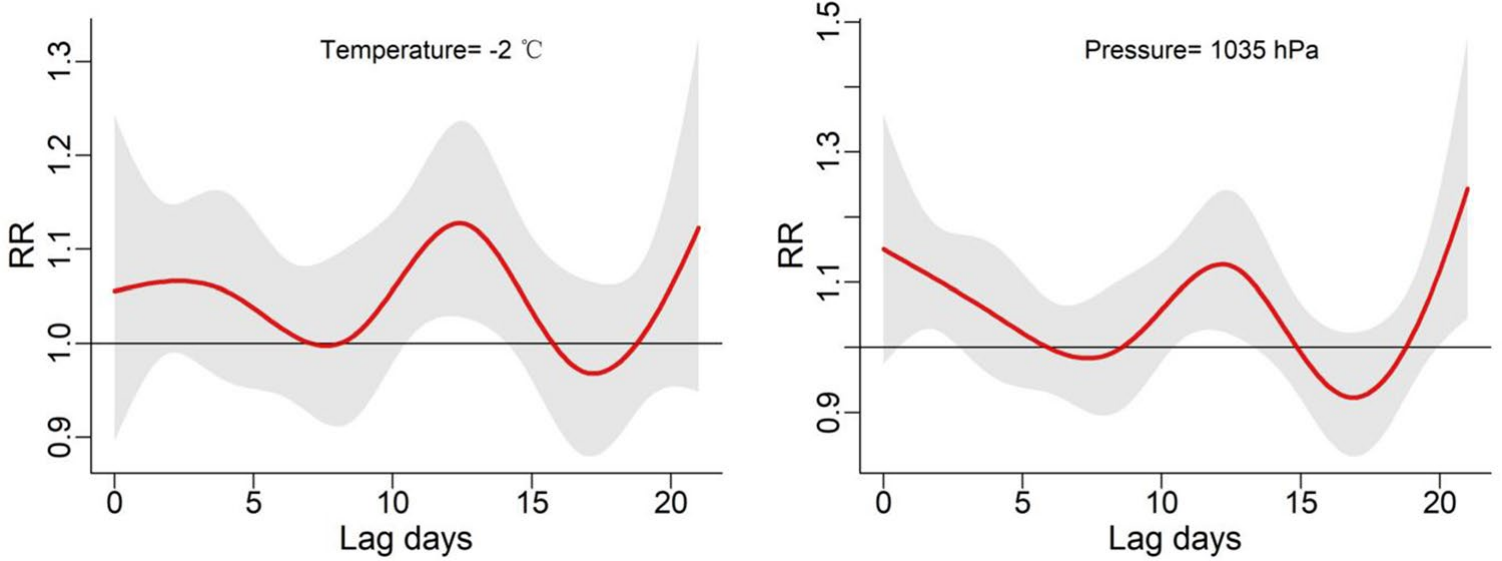
The effect of temperature and air pressure on Bell's palsy at different time lags under − 2 °C and 1035 hPa conditions.

**Figure 7:**
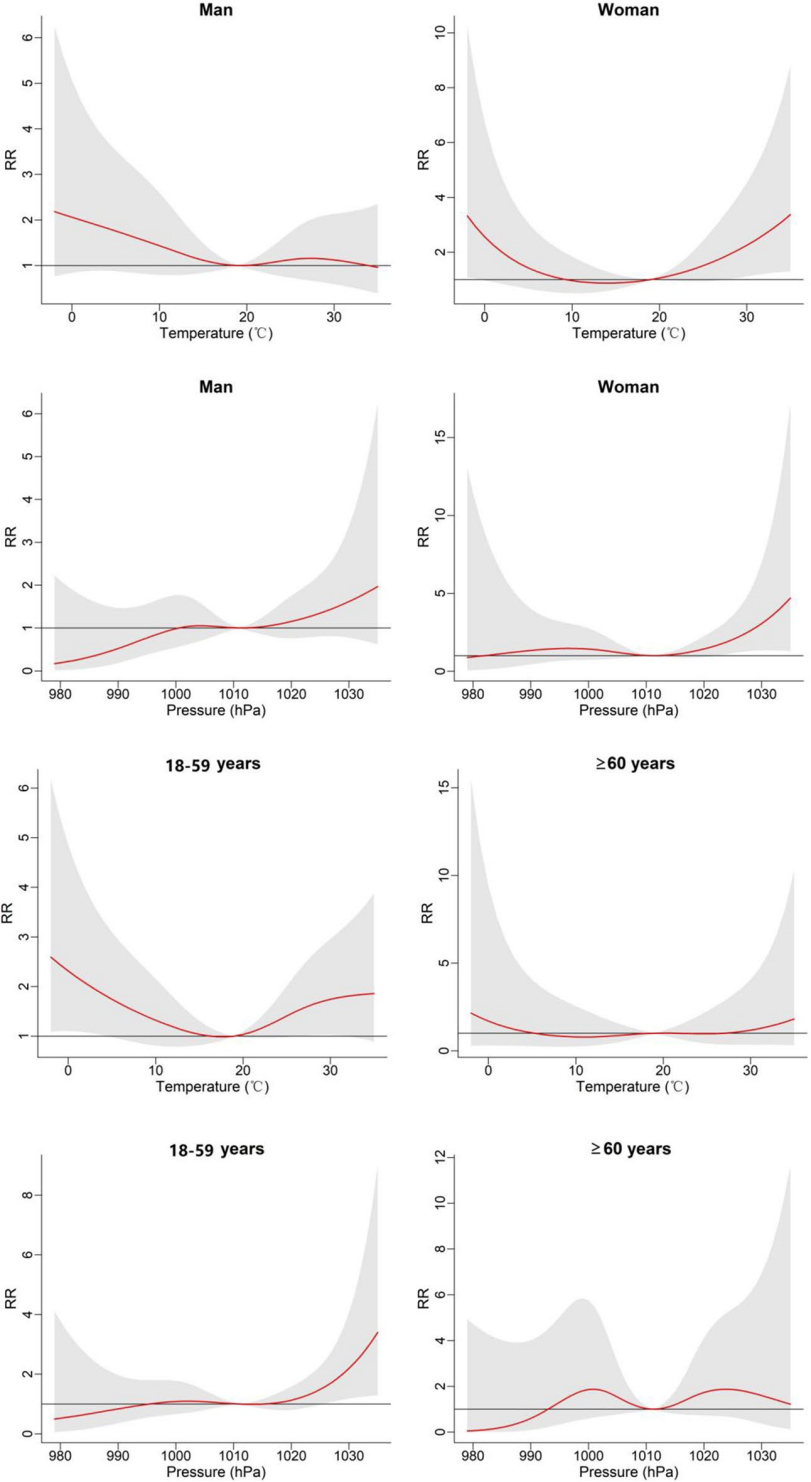
The overall effect of temperature and air pressure on Bell's palsy in different sex and age groups.

To gain deeper insights into the lag effect of meteorological factors, we incorporated temperature and air pressure into the DLNM. The results are visually depicted through 3D plots in Fig. 4, illustrating the exposure-lag-response relationship between temperature, air pressure, and the onset of BP. At Lag = 0, the relative risk (RR) of BP onset was most pronounced at both low and high temperatures. Low temperatures maintained a relatively elevated risk for onset, whereas high temperatures exhibited a diminishing risk of onset with prolonged lag periods. Furthermore, high air pressure displayed a higher relative risk compared to low pressure and became more significant with longer lag days. Figure 5 shows the overall effect of meteorological factors on BP onset. The overall effect curve of temperature is U-shaped and the curve for air pressure resembles an S-shaped. Temperature exhibited statistical significance within the range of − 2 to 3 °C, with the peak occurring at − 2 °C (RR = 2.66, 95% CI 1.22—5.80). Similarly, air pressure within the range of 1022 to 1035 hPa showed significant differences, the effect was strongest at 1035 hPa (RR = 2.90, 95% CI 1.21–6.94). To achieve a clearer comprehension of the impact of time lags on BP, Fig. 6 illustrates the effects of meteorological factors on BP at different time lags under low temperature and high air pressure conditions. The impact of both factors follows a W-shaped curve, with the maximum peak occurring at lag = 12 (RR = 1.13, 95% CI 1.03–1.23) at a temperature of − 2 °C. At air pressure of 1035 hPa, the maximum peak occurs at lag = 21 (RR = 1.24, 95% CI 1.05–1.48). Subgroup analyses (Fig. 7) revealed heightened susceptibility to low temperature and high air pressure environments among females and young to middle-aged individuals.

In addition, the dataset consisting of records from 1763 hospitalized patients documented the precise onset of their illnesses, including the triggers of BP. The triggering factors for the onset of illness, in descending order, include exposure of the face to windy or cold conditions, fatigue, and nocturnal sleep deprivation. The prevalence of BP associated with exposure to cold or windy conditions during the spring to winter seasons stood at 30.89, 35.93, 33.51, and 35.80% of the overall count of hospitalizations, respectively, which was not a significant difference (*p* = 0.609).

## Discussion

The etiology and pathogenesis of BP remain unclear. Although BP is generally considered a self-limiting condition, up to 30% of afflicted patients may not achieve full recovery or may experience severe sequelae adversely impacting their life^3^. This study aspires to elucidate the true relationship between meteorological factors and the incidence of BP. Such insights hold significant implications for the prevention of BP preceding its onset, the preservation of facial nerve function following its occurrence to enhance treatment efficacy or reduce sequelae.

The investigation of the seasonal correlation of BP onset has been extensively explored in the existing literature. In our study, we observed that BP outbreaks were most prevalent during the autumn season, exhibiting a higher occurrence during autumn and winter compared to spring and summer. This is consistent with the outcomes of a Korean study, which concluded that a significant temperature drop during autumn had a more pronounced impact on BP outbreaks than cold temperatures during winter^11^. Nevertheless, we acknowledge that categorizing the disease by seasons possesses certain limitations due to the limited differentiation of meteorological factors between the seasons. Our study results suggest that a monthly breakdown provides a more nuanced representation of the interplay between meteorological factors and disease incidence. Although BP cases in our study exhibited a higher incidence in autumn, it is noteworthy that this elevated incidence coincided with months characterized by lower temperatures, thus supporting the hypothesis that low temperatures may constitute a risk factor.

In our study, the occurrence of BP during the summer months was not infrequent, and the number of BP triggered by exposure to cold or windy conditions in the summer ranked second only to that in winter. We posit that, despite the prevailing high temperatures during the summer season, the common use of indoor air-conditioning in Hangzhou creates a notable temperature differential between indoor and outdoor environments. It has been proposed that prolonged exposure to air-conditioning during the summer months may lead to reduced levels of vitamin D and melatonin, potentially impacting immune system function^21^. Autoimmunity is also considered to be associated with the development of BP^2^. Those indirect evidences support that the utilization of indoor air-conditioning in summer may heighten the risk of developing the disease. While DLNM model demonstrated high temperature as a risk factor for BP onset at lag = 0, the univariate analysis of temperature did not yield statistical significance regarding its overall effect on onset. This observation needs further investigation for validation.

Although this study is not the first to investigate the correlation between meteorological factors and the onset of BP, it stands out for the inclusion of a substantial number of cases and its focused examination within a geographically confined and small area. These factors contribute to the generation of more authentic evidence for the examination of this issue. Through a comparative analysis of temperature, air pressure, and case distribution, we observed that the incidence of BP exhibited a similar pattern to the mean air pressure, while demonstrating an inversely proportional relationship with the mean temperature. This finding aligns with the results of two prior studies^8,10^. Furthermore, combined with the outcomes derived from our established DLNM, we identified low temperature and high air pressure as risk factors for the development of BP, with a certain time lag. A parallel study has previously indicated that mean temperature may influence the course of BP, while mean air pressure is correlated with the severity of clinical symptoms associated with BP^16^. Additionally, another investigation suggested that prolonged periods of high air pressure may elevate the risk of BP^17^. Nonetheless, the results of other studies do not uniformly support this conclusion^14,18^.

Drawing upon theories and research findings, we believe that low temperature and high air pressure constitute influential factors in BP. Low temperature can manifest in various scenarios, including prolonged cold exposure during winter, significant temperature drops in autumn, or substantial temperature differentials between indoor and outdoor environments in summer. Some scholars share a similar view and suggest that cold stimulation can lead to the change of secretory factors from fat cells increasing the risk of BP, and it is also believed that low temperature tends to change the microenvironment of facial microvascular neuron^1^. It may induce peripheral vasoconstriction, potentially resulting in microvascular ischemia of the facial nerve and subsequent morbidity, with a more pronounced effect in diabetic patients^17^. Furthermore, some studies have proposed that the association between BP and hypothermic seasons may be linked to heightened activation of the HSV-1 virus^12,22^. Regarding the influence of high air pressure, it has been postulated that an increase in atmospheric pressure may elevate middle ear pressure, potentially amplifying the risk of morbidity^17^. High air pressure has demonstrated an impact on certain vascular diseases^23,24^, and it may also contribute to the pathogenesis of BP by influencing vasoconstriction processes. Subgroup analyses unveiled that females and young and middle-aged patients with BP, exhibited heightened sensitivity to temperature and air pressure. Similar investigations into various diseases and meteorological factors have indicated that women and the elderly tend to be more responsive to environmental changes^25–27^. In the case of the elderly, we hypothesize that their greater propensity to remain indoors and reduce outdoor activities during extreme weather conditions contributes to their decreased risk, as they have fewer hours of exposure to weather extremes. However, subgroup analyses may be limited by reduced case numbers, necessitating further evidence to substantiate these findings.

While this study encompassed a dataset of 5387 BP cases collected over a 5-year period, it has some potential limitations. The entirety of BP patient data was sourced from a single medical center, and due to the exclusion of cases with incomplete data, it may not fully represent the complete BP incidence in Hangzhou. Additionally, due to the study period coinciding with the COVID-19 pandemic, the incidence of BP was affected by the pandemic^28^, potentially linked to the known association of the novel coronavirus with facial nerve involvement^29^. Some reports indicate that COVID-19 vaccination has increased the incidence of BP^30^. Moreover, during the COVID-19 pandemic, patients experienced heightened negative emotions^31^, along with specific treatment limitations that were unfavorable for BP recovery. Future research should delve deeper into the specific relationship between COVID-19 and BP.

## Conclusion

This study suggests that low temperature and high air pressure serve as risk factors for the onset of BP with a certain time lag. Implementing preventive measures to mitigate the risks associated with exposure to low temperatures and high-pressure environments has been empirically validated as advantageous. It is imperative for individuals to remain vigilant regarding daily weather fluctuations, both in the interest of averting the onset of BP and for safeguarding the facial nerve following its occurrence. Protective measures may encompass reducing outdoor exposure and limiting the duration of outdoor activities during inclement weather, alongside ensuring adequate warmth for the head and face. Patients with BP are also advised to exercise discretion regarding engagement in high-pressure environmental activities, such as air travel or diving, when circumstances permit. Furthermore, we contend that exploring the correlation between specific months and the onset of BP offers a more dynamic perspective and better reflects real-world outcomes compared to analyzing solely based on seasonal correlations.

### Supplementary Information

Supplementary Figure 1.

## Data Availability

The datasets analysed during the current study are available from the corresponding author on reasonable request. However, individual de-identified participant data is not shared.
