# Supplementary figures and images for: Effect of temperature and air pressure on the incidence of Bell's palsy in Hangzhou: a distributed lag non-linear analysis
Source: Sci Rep. 2023 Nov 22;13:20424. doi: 10.1038/s41598-023-47570-2 (PMC10665392; doi:10.1038/s41598-023-47570-2)

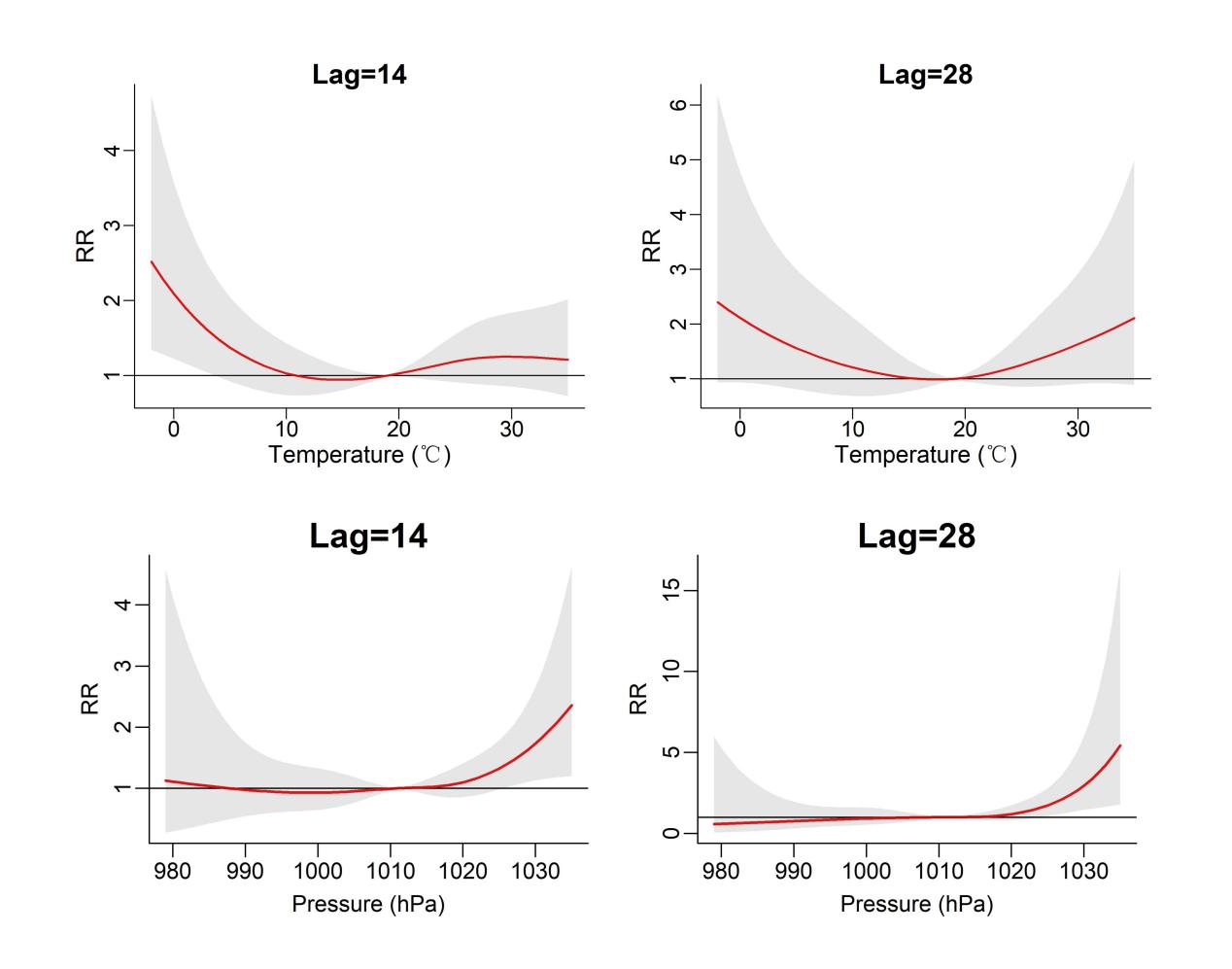


Figure S1.The overall effect of temperature and air pressure on Bell's palsy at lag 0-14 and lag 0-28.

Supplement: Supplementary file 1 — Supplementary Figure 1. [file 41598_2023_47570_MOESM1_ESM.docx]
